# Synthetic viruses—Anything new?

**DOI:** 10.1371/journal.ppat.1007019

**Published:** 2018-10-04

**Authors:** Volker Thiel

**Affiliations:** 1 Department of Infectious Diseases and Pathobiology, University of Bern, Bern, Switzerland; 2 Federal Department of Home Affairs, Institute of Virology and Immunology, Bern and Mittelhäusern, Switzerland; University of Pittsburgh, UNITED STATES

## Synthetic horsepox virus raises concerns

The report of the construction of an infectious horsepox virus from synthesized DNA by Noyce, Lederman, and Evans [1] raised considerable concerns about whether this study will facilitate the construction of smallpox virus (variola) using synthetic biology [2–5]. This is a valid concern, but for a number of reasons—as explained below—no major change concerning the likelihood of a “resurrection” of smallpox emerges from this publication. Having said this, it is also evident that the scientific community, politicians, decision makers, and the lay public have to continue, and probably intensify, a discussion on benefits and risks of synthetic biology in a broader sense.

## What has been done?

The study “Construction of an infectious horsepox virus vaccine from chemically synthesized DNA fragments” was conducted in the laboratory of Dr. Evans, University of Alberta, Canada. The authors describe a workflow from chemically synthesized DNA to the rescue of infectious horsepox virus with the intention to generate a horsepox virus–based vaccine that may be equally as efficacious as vaccinia virus–based vaccines but may have less severe side effects. Since the introduction of vaccinia virus as a vaccine against smallpox by Edward Jenner more than 200 years ago, the origin and passage history of this vaccine have remained elusive. However, recent sequencing data revealed close similarities between horsepox and vaccinia viruses, suggesting that horsepox virus might serve as a candidate smallpox vaccine [6–8]. There is just one problem: there have been no horsepox virus infections reported since the 1980s, and the virus might actually be extinct [9]. Therefore, Evans and colleagues set out to “resurrect” horsepox virus from synthesized DNA. To achieve this, they made use of (i) the ability of poxviruses to facilitate homologous recombination, (ii) a helpervirus (Shope fibroma virus) to launch poxvirus replication from naked DNA [10], and (iii) recent advances in synthetic biology, namely, the ability to chemically synthesize large DNA fragments.

## Anything new?

**Fig 1:**
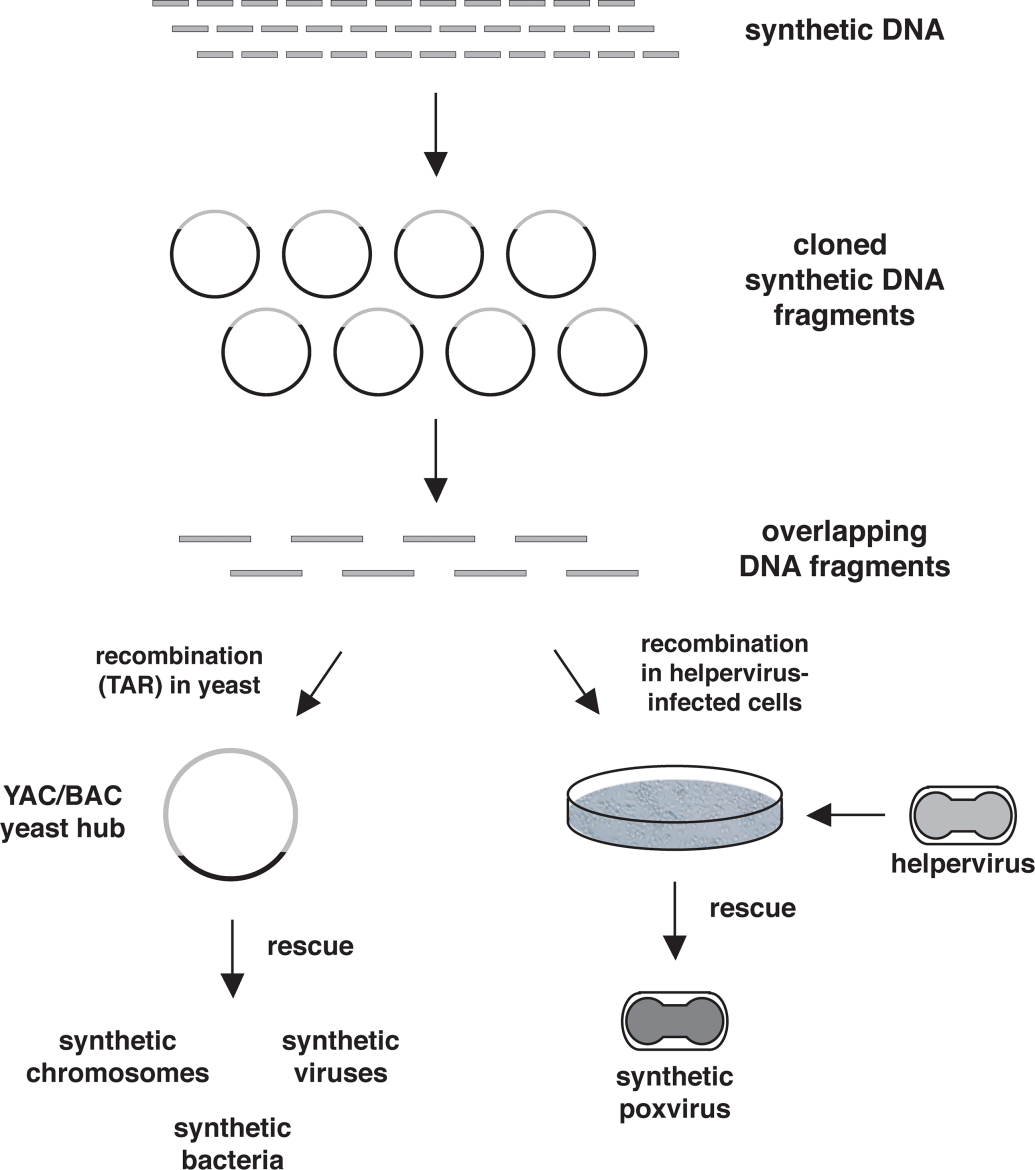
Cloning of synthetic DNA. The generation of synthetic poxviruses as described by Evans and colleagues and cloning of synthetic DNA using TAR in yeast are illustrated. Synthesized DNA fragments are assembled and cloned in a set of plasmids containing overlapping DNA fragments. Release of cloned DNA fragments from plasmids creates a set of overlapping DNA fragments that can recombine in yeast (TAR cloning) to form a YAC/BAC (left side) or in helpervirus-infected cells to rescue poxviruses (right side). The yeast hub is versatile and allows for the generation of synthetic viruses, bacteria, and even eukaryotic chromosomes. BAC, bacterial artificial chromosome; TAR, transformation-associated recombination; YAC, yeast artificial chromosome.

The authors are now blamed for providing a workflow that allows for the generation of any infectious poxvirus, including smallpox virus, from synthesized DNA (Fig 1). But is this actually new? According to the PLOS Dual Use Research of Concern (DURC) Committee, “The study did not provide new information specifically enabling the creation of a smallpox virus, but uses known methods, reagents and knowledge that have previously been used in the synthesis of other viruses (such as influenza and polio viruses)” [5]. Indeed, the individual experimental steps and methods to generate infectious horsepox virus have been reported before.

First, it has long been known that poxviruses can mediate homologous recombination, and this has been used for decades to modify mainly vaccinia virus, but also other poxviruses. It is therefore not surprising that overlapping DNA fragments are joined in poxvirus-infected cells by homologous recombination [10–12].

Second, procedures to rescue poxviruses from naked DNA have also been established for decades [13, 14]. Helperviruses, such as fowlpox virus, are routinely used to launch replication from naked vaccinia virus genomic DNA. This procedure has been successfully used to generate recombinant vaccinia viruses containing insertions of up to 26–31 kbp of foreign DNA [15, 16]. Moreover, fowlpox virus has also been used as a helpervirus to launch vaccinia virus replication from a full-length vaccinia virus DNA cloned as a bacterial artificial chromosome (BAC) in *Escherichia coli* [17]. This work by Domi and Moss is remarkable because it demonstrated for the first time that infectious vaccinia virus has been obtained from cloned circular DNA as opposed to previous techniques that required a linear vaccinia virus genome with authentic genome ends.

Third, it comes as no surprise that it is possible to generate infectious viruses by using synthesized DNA fragments [18]. The first synthetic virus, poliovirus, was produced by Wimmer and colleagues and made us aware of the fact that we entered a new era of reverse genetics that allows for the generation of synthetic viruses without the need for a nucleic acid template [19]. This is instrumental to generate infectious viruses for which no isolates are available. The 1918 “Spanish” influenza virus is the first example of a “resurrected” virus that was constructed by only knowing the genome sequence [20]. Also, more complex and larger RNA viruses, such as coronaviruses (up to 30 kb genome size), can be synthesized, as demonstrated by Denison and colleagues for a Severe Acute Respiratory Syndrome (SARS)-like virus [21]—a virus that was sequenced from bat samples and represents the likely origin of the SARS coronavirus that caused an epidemic starting in China in 2002.

## Synthetic biology—Quo vadis?

The synthesis of infectious horsepox virus by Evans and colleagues demonstrates that synthetic biology has entered the field of large DNA viruses. Although the procedure to generate synthetic horsepox virus by Evans is quite specific for poxviruses, very general and widely applicable procedures to assemble and clone large DNA fragments using transformation-associated recombination (TAR) in yeast (*Sacharomyces cerevisae*) have been established. By using overlapping synthetic DNA fragments, TAR cloning, and yeast as a hub, it was possible to clone full-length herpesvirus genomes (human cytomegalovirus [hCMV] and herpes simplex virus 1 [HSV1]) as yeast artificial chromosomes (YACs) [22, 23]. The YACs have been transferred into *E*. *coli* for DNA amplification in the form of a BAC, and synthetic hCMV and HSV1 were rescued following transfection into appropriate mammalian cells. This procedure is versatile (Fig 1), and it is equally applicable to assemble and clone poxvirus genomes. Moreover, a YAC/BAC carrying a poxvirus genome can be used to launch replication of infectious poxviruses as described by Domi and Moss [17].

These examples illustrate the fact that synthetic biology has matured towards a powerful technique that will impact the scientific community—and our society in general—similar to the advent of recombinant DNA technology in the 1970s. It is already possible to generate synthetic bacteria [24–26] and eukaryotic chromosomes [27–34], and it can be expected that synthetic eukaryotic cells will follow soon. Therefore, the impact of synthetic biology goes far beyond the question of DURC, as in the case of viruses, and we have to find a way to cope with the fact that this technology will allow the generation of designer microbes and, ultimately, synthetic life.

In Switzerland, a discussion has been initiated by the Swiss Academy of Sciences that developed as a result of workshops with life scientists from Swiss academic institutions on ways of addressing the misuse potential of biological research [35]. Similar initiatives in which benefits and risks of DURC can be openly discussed have been launched in many countries and are needed to raise awareness within the scientific community. However, it should be noted that we have sufficient regulations in place to ensure biosafety and biosecurity. Moreover, we have already seen the benefits of synthetic viruses. The recovery of the pandemic 1918 influenza virus provided important mechanistic insight into critical determinants of virus tropism, transmission, and pathogenicity. Likewise, the recovery of the SARS-like bat coronavirus shed light on determinants of zoonotic infection. Such information is urgently needed to understand cross-species transmission of contemporary virus strains and to assess the risk of emerging pandemic viruses. Synthetic viruses also allow us to explore novel concepts to combat virus infection, such as virus attenuation by large-scale recoding, pioneered by Wimmer and colleagues [18]. It is foreseeable that such concepts will greatly increase our preparedness to emerging viruses because it is now just a matter of weeks to generate synthetic viruses from genomic sequences and at the same time to synthesize attenuated candidate vaccine strains. The fast technological advances in synthetic biology illustrate that the breadth of synthetic biology goes beyond DURC, and our discussions should be well balanced to allow this novel technology to evolve. We’re just beginning to explore the potential of synthetic biology that is expected to become a powerful tool to reveal groundbreaking insights in all fields of life sciences.
